# Perspectives on the Treatment of Multiple Myeloma

**DOI:** 10.1093/oncolo/oyad306

**Published:** 2023-11-23

**Authors:** Abdul Rafae, Frits van Rhee, Samer Al Hadidi

**Affiliations:** Department of Hematology and Oncology, University of Arkansas for Medical Sciences, Little Rock, AR, USA; Myeloma Institute, Winthrop P. Rockefeller Cancer Institute, University of Arkansas for Medical Sciences, Little Rock, AR, USA; Myeloma Institute, Winthrop P. Rockefeller Cancer Institute, University of Arkansas for Medical Sciences, Little Rock, AR, USA

**Keywords:** multiple myeloma, treatment, transplantation

## Abstract

The treatment of multiple myeloma has evolved significantly over the past few decades with the development of novel therapeutics. The introduction of proteasome inhibitors, immunomodulatory drugs, monoclonal antibodies, and high-dose chemotherapy followed by hematopoietic stem cell transplantation has led to improved response rates and survival outcomes. The use of bispecific antibodies and chimeric antigen receptor T-cell therapy is currently under study, and early results are showing promise. Although outcomes for patients with MM have improved with the development of new treatments, there remains a subset of patients with high-risk disease who have a particularly poor prognosis. Therefore, it is critical that future clinical trials focus on developing new therapies specifically for high-risk multiple myeloma. Here we review the literature and provide guidance on treating patients with multiple myeloma for practicing oncologists.

## Teaser

Although outcomes for patients with multiple myeloma have improved with the development of new treatments, there remains a subset of patients with high-risk disease who have a particularly poor prognosis. This article reviews the literature and provide guidance for the practicing oncologist on treating patients with multiple myeloma.

**Implications for Practice**

The evolving treatment options for multiple myeloma have significant implications for clinical practice. With the availability of newer therapeutics, clinicians must stay up to date with the latest treatment options and incorporate them into their practice. As treatment options continue to evolve, we describe our approach to treating multiple myeloma patients for practicing oncologists.

## Introduction

Multiple myeloma (MM) is a type of hematological malignancy that affects the plasma cells in bone marrow.^1^ According to the American Cancer Society, an estimated 35 730 new cases of MM will be diagnosed in the US in 2023, and ~12 590 deaths will be attributed to the disease.^2^ The incidence of MM increases with age, with a median age at diagnosis of 69 years. Men are slightly more likely to develop MM than women.^2^ African Americans have a higher incidence and mortality rate compared to other racial/ethnic groups and continue to face disparities that limit appropriate management and affect outcomes negatively.^3-9^ Early diagnosis and effective treatment are critical in improving outcomes for patients with MM.

Over the years, significant advancements have been made in the treatment of MM, resulting in improved outcomes and quality of life for patients.^10-13^ Traditional chemotherapy and radiation remain important treatment modalities, but newer therapeutics, including proteasome inhibitors (PIs), immunomodulatory drugs (IMiDs), and monoclonal antibodies, have shown promising results and are currently incorporated in the treatment paradigm.^14^ The use of high-dose chemotherapy and tandem hematopoietic stem cell transplantation along with the introduction of PIs and IMiDs resulted in long term survival with an approximately one-third of patients achieving cure.^10,12^ Despite these advances, challenges such as treatment-related adverse events (AEs), drug resistance and relapse still exist, highlighting the need for future research to develop more effective and safer therapies.^15^ In this context, this paper will explore the current state of treatment options for MM, their limitations, and future perspectives.

## Biology of Multiple Myeloma

MM is a complex and heterogeneous disease with diverse biological features.^16-19^ Frequent chromosomal abnormalities along with several other genetic mutations have been identified that contribute to the development and/or progression of MM.^18,19^ These mutations can affect signaling pathways involved in the growth, survival, and migration of myeloma cells. For example, mutations in the KRAS and NRAS genes can activate the MAPK/ERK pathway, promoting cell proliferation and survival.^20-22^ Deletion of the TP53 tumor suppressor gene and/or mutations in TP53 is associated with poor prognosis and treatment resistance.^23-25^

The bone marrow microenvironment also plays a crucial role in the biology of MM.^26,27^ Myeloma cells interact with various cells in the bone marrow, including stromal cells, osteoclasts, and immune cells, through cell adhesion molecules and cytokines. This interaction promotes the growth and survival of myeloma cells and leads to bone destruction and immune dysfunction. The dysregulation of the Wnt signaling pathway and upregulation of DKK1 promote osteoclastic activity, resulting in the formation of lytic bone lesions in MM.^28^

Furthermore, other dysregulations occur in epigenetics, such as hypo or hyper-methylation of certain genes, which contribute to the disease pathogenesis.^29^ The bone marrow microenvironment also contributes to treatment resistance by providing a protective niche for myeloma cells.^30^

Immunologically, MM is characterized by a reduction in the diversity and function of T cells and natural killer (NK) cells, as well as an increase in regulatory T cells and myeloid-derived suppressor cells.^31^ This immune dysfunction can contribute to disease progression and treatment resistance and makes MM patients more susceptible to infections.^32^

## Risk Stratification

**Figure 1.**
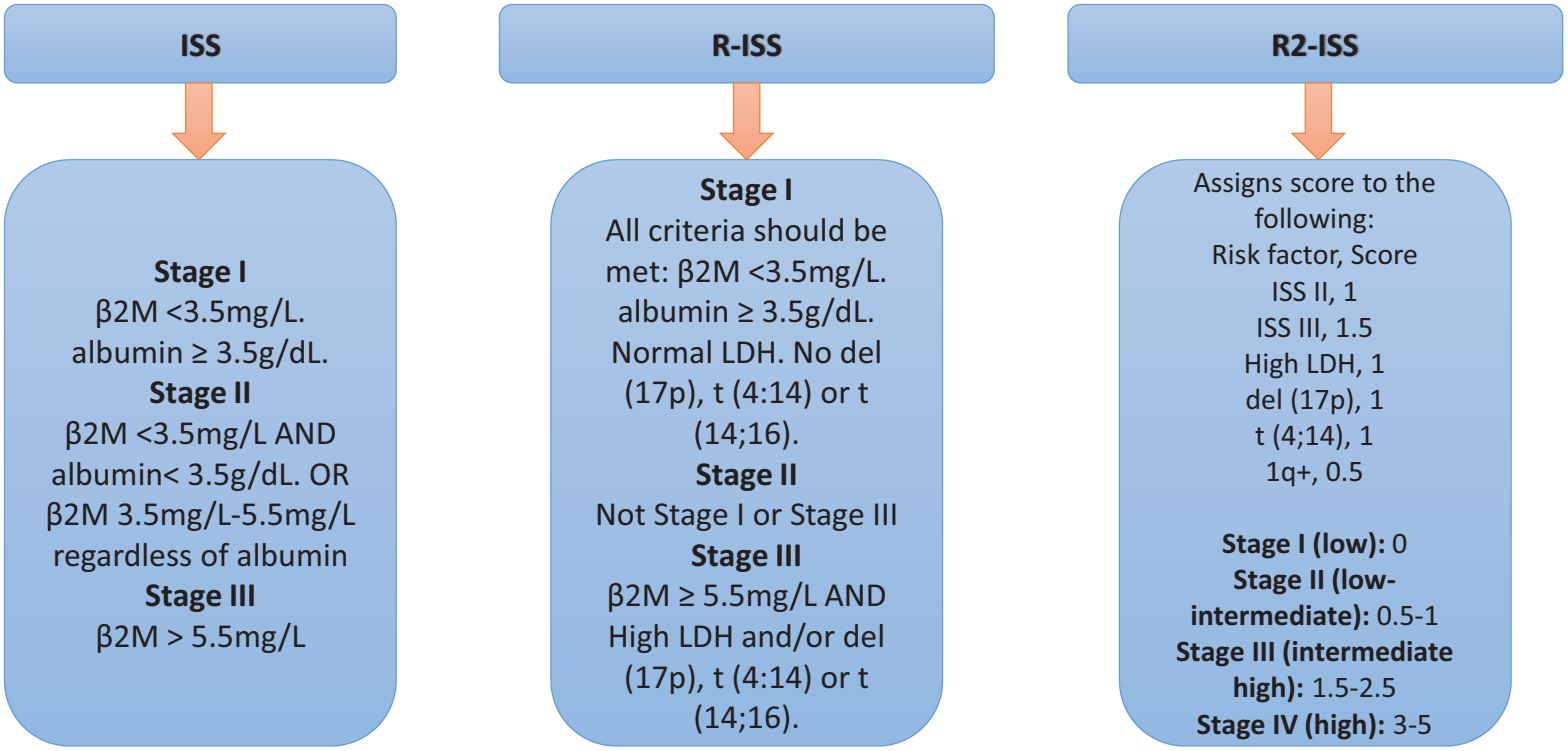
Multiple myeloma staging: evolution of International Staging System (ISS).

The survival of patients with MM is associated with several key factors, including the extent of the tumor burden, specific cytogenetic characteristics, and the response to treatment. To categorize patients based on these factors, various tools and scoring systems are used in medical practice (Fig. 1).

Abnormalities involving Chromosome 1q, such as gain or amplification, hold particular significance due to their association with unfavorable outcomes. Despite being observed in as many as 40% of newly diagnosed MM patients, the exact mechanism behind their influence on prognosis remains poorly understood. A more recent development is the revised version of the Revised International Staging System (R-ISS) from the European Myeloma Network, which now incorporates the presence of 1q + abnormalities due to their demonstrated prognostic relevance.^33^ This updated system also takes into consideration that having more than one high-risk cytogenetic abnormality compounds the negative prognosis. More data is needed to better understand the effects of 1q + abnormalities especially in the presence and/or absence of other cytogenetic abnormalities. Additionally, the presence of 3 or more focal bone lesions is indicative of an unfavorable prognosis.^34^

Several other factors also serve as poor prognostic indicators, including renal failure, the quantity of circulating plasma cells, the presence of plasma cell leukemia, and the occurrence of extramedullary disease.^35^ In conjunction with the R-ISS, it is imperative to account for all these factors, particularly when classifying myeloma as high risk.

## Treatment of Multiple Myeloma

The treatment of MM typically involves several stages, including induction therapy, consolidation therapy, and maintenance/extended therapy. Induction therapy involves the use of several drugs to achieve a deep response, followed by consolidation therapy to further reduce disease burden. Maintenance/extended therapy is then used to help sustain and deepen remission and prevent disease relapse.

The specific drugs and treatment regimens used in each stage of treatment may vary based on the patient’s age, overall performance status and health condition, disease characteristics and eligibility for high-dose chemotherapy and hematopoietic stem cell transplantation (HSCT).

In addition to drug therapy, supportive care measures are also an important aspect of the management of MM. These may include treatments to manage pain, prevent infections and/or thrombosis, decrease risk of pathological fractures and address other symptoms associated with the disease.^36-39^ Overall, the goal of treatment for MM is to achieve a deep and sustained response while minimizing treatment-related toxicity and improving quality of life.

## Transplant Eligibility

The eligibility for autologous HSCT depends on multiple factors including patient’s age, overall health and disease status and may vary from institution to institution and across different countries. We recommend evaluation at a transplant center to determine eligibility for all patients with MM regardless of age since the decision to proceed with autologous HSCT requires detailed discussion with patient and special consideration to patient and disease characteristics, along with the response to previous treatments.

A frailty score that combines age, functional status, and comorbidities can predict survival and treatment-related toxicity and is useful to determine the feasibility of a treatment regimen.^40^ While younger patients may have more treatment options without concerns about fitness, older adults may be more likely to be over- or under-treated due to their physical condition. To assist in decision-making, objective and straightforward ways to evaluate a patient’s fitness and ability to tolerate therapy will be increasingly important. Studies have consistently shown that patients categorized as less fit or frail are at higher risk of inferior outcomes and increased toxicities, highlighting the importance of accurately assessing fitness.^41^

Autologous HSCT has been shown to improve survival in MM patients. Upfront autologous HSCT in transplant-eligible patients is the current standard of care. Studies have shown that upfront autologous HSCT leads to better progression-free survival (PFS) compared to delaying autologous HSCT until relapse, but there is no significant difference in overall survival (OS).^42,43^ The choice of timing should be based on various factors such as disease risk, age, comorbidities, and disease progression. For high-risk MM patients, upfront autologous HSCT within 12 months of diagnosis offers the best chance of achieving a deep response, particularly minimal residual disease (MRD)-negative status, which is associated with superior PFS and OS.^44^ It is important to consider the potential risks of delayed HSCT, such as worsening performance status, comorbidities, and aggressive disease relapse, which may render patients ineligible for transplantation. A retrospective study found that there is an approximate 12% risk of becoming transplantation ineligible when delaying ASCT.^45^ Therefore, stem cells should be collected within 4 to 6 months of diagnosis in all transplantation-eligible patients to ensure that this treatment option is available if needed in the future. Overall, the timing of autologous HSCT should be individualized based on the patient’s-specific circumstances, and upfront autologous HSCT should be considered for high-risk MM patients to achieve the best outcomes.^46^ It is our recommendation to proceed with upfront autologous HSCT in transplant eligible patients and consider tandem autologous HSCT in patients with high-risk MM based on long-term survival benefit based on long-term follow-up from total therapy protocols done at the University of Arkansas for Medical Sciences.^10^

## Newly Diagnosed Multiple Myeloma

When choosing induction therapy for MM, various factors need to be taken into consideration. The patient-related factors include age, fitness level, caregiver support, comorbidities, compliance, and lifestyle preferences. Disease-related factors include ISS (International Staging System)/R-ISS, presence of extramedullary disease, number of bone lesions on advanced imaging, organ functions, and molecular cytogenetics. Treatment-related factors include access to standard-of-care therapies, costs and copays, route of administration, clinical trial availability, and toxicity. It is important to evaluate all of these factors in order to provide a personalized treatment approach that balances efficacy and safety and provides the best possible outcome for the patient.^47^ Our approach for treatment newly diagnosed MM depends on transplant eligibility.

## Transplant Eligible

Induction treatment for transplant eligible newly diagnosed MM should include a combination therapy of 3-4 drugs including an IMED, a PI, steroid with or without anti-CD38 antibody. The use of RVD (lenalidomide, bortezomib, and dexamethasone) is supported by the results of SWOG S0777 phase III clinical trial.^48,49^ A long-term follow-up analysis included 460 patients with median follow up of 84 months. Median PFS was 41 months for RVD and 29 months for RD, with a statistically significant hazard ratio of 0.74. Median OS was not reached for RVD and was 69 months for RD, with a statistically significant hazard ratio of 0.71. RVD demonstrated better PFS and OS than RD and was found to be an appropriate standard of care for newly diagnosed MM regardless of age. To minimize toxicity, the low-dose dexamethasone regimen (40 mg once a week) is preferred in all regimens. It is our practice to decrease the dose of dexamethasone to 20 mg once a week for most elderly patients (≥75 years). In a randomized trial by the Eastern Cooperative Oncology Group (ECOG), the low-dose dexamethasone approach was associated with superior OS and significantly lower toxicity.^50^

Data from the phase III DETERMINATION trial and IFM 2009 study showed that HSCT provided median PFS benefit without significant difference in OS.^42,51^ However, both studies included predominantly standard risk cytogenetics (Table 1).

**Table 1.** Selected treatment regimens for transplant eligible newly diagnosed multiple myeloma.

Clinical trial	Number of patients	Treatment regimen	Median age (years)	Median follow up (months)	Progression-free survival (months)
*4 drug vs 3 drugs*					
GMMG-HD7^52^	660	Isa-RVD vs. RVD	59	4.2	N/A
GRIFFIN^53,54^	207	Dara-RVD vs. RVD	60	49.6	NR for both groups
CASSIOPEIA^55^	1085	Dara-VTD vs. VTD	58.5	35.4	NR vs. 46.7
*3 drug combinations*					
DETERMINATION^51^	357	RVD vs RVD + HSCT	57 vs 55	76	46.2 vs 67.5
Myeloma XI^56^	2042	CRD-HSCT vs CTD-HSCT	61	36.3	36 vs 33
FORTE^57^	474	KRd-HSCT vs KCd-HSCT	57	50.9	NR vs 53
IFM-DFCI^42^	700	RVD-HSCT vs RVD	59	43	50 vs 36

Abbreviations: Isa-RVD, isatuximab, lenalidomide, bortezomib, dexamethasone; Dara-RVD, daratumumab, lenalidomide, bortezomib, dexamethasone; Dara-VTD, daratumumab, bortezomib, thalidomide, dexamethasone; RVD, lenalidomide, bortezomib, dexamethasone; HSCT, hematopoietic stem cell transplant; CRD, cyclophosphamide, lenalidomide, dexamethasone; CTD; cyclophosphamide, thalidomide, dexamethasone; KRd, carfilzomib, lenalidomide, dexamethasone; KCd, carfilzomib, cyclophosphamide, dexamethasone; HSCT, hematopoietic stem cell transplant; NR, not reached; N/A, not available.

Bortezomib therapy was initially associated with significant peripheral neuropathy (PN). The neuropathy can be severe and painful, but the risk can be reduced by administering the drug subcutaneously once a week instead of intravenously twice weekly.^58,59^ The ENDURANCE trial compared KRD (carfilzomib, lenalidomide, dexamethasone) to RVD. The study found that the KRD regimen did not improve PFS compared to RVD and had more grade III/IV toxicity.^60^ In the RVD group, out of 527 patients, 44 (8%) experienced PN, 9 (2%) experienced dyspnea, and 11 (2%) experienced thromboembolic events. In the KRD group, out of 526 patients, 4 (<1%) experienced PN, 38 (7%) experienced dyspnea, and 26 (5%) experienced thromboembolic events. Treatment-related deaths occurred in 2 patients (<1%) in the RVD group (one due to cardiotoxicity and one due to secondary cancer) and in 11 (2%) in the KRD group (4 due to cardiotoxicity, 2 due to acute kidney failure, one due to liver toxicity, 2 due to respiratory failure, one due to thromboembolic event, and one due to sudden death) (Table 1).

It is important to note that ENDURANCE trial included patients with standard risk disease since the trial excluded patients with del17p, t(14;16), t(14;20), plasma cell leukemia, or lactate dehydrogenase (LDH) > 2 times upper limit of normal. The use of carfilzomib in high-risk disease may provide superior outcomes; however, the potential benefit of carfilzomib in newly diagnosed MM patients is supported by single-arm studies and/or retrospective studies. In our experience, we offer carfilzomib-based regimens for patients with high-risk MM given the potential benefit and lack of improvement in prognosis of high-risk patients when treated with bortezomib-based regimens.^10^

FORTE trial showed that KRd with HSCT outperformed the other 2 treatments (KRD) with no HSCT and carfilzomib plus cyclophosphamide plus dexamethasone (KCD). Additionally, maintenance therapy using carfilzomib plus lenalidomide improved PFS compared to standard lenalidomide alone.^57^ Compared to ENDURANCE trial, non-hematological AEs were lower. Also, when combined with HSCT, KRD showed better PFS (median NR vs 47 months in IFM 2009); however, this cross-trial observation will need a proof by randomized controlled trial prior to any definite conclusion.

The addition of anti-CD38 antibody to triplet regimens (ie, a quadruplet regimen) resulted in deeper responses and is currently being used more frequently. One randomized study showed that daratumumab in combination with bortezomib, thalidomide, and dexamethasone (Dara-VTd) had higher response rates, PFS, and a tendency toward better OS compared to VTd.^61^ CASSIOPEIA study enrolled a total of 1085 patients who were randomly assigned to receive either Dara-VTd (*n* = 543) or VTd (*n* = 542).^55^ In the intention-to-treat population, at day 100 after transplantation, 157 (29%) of 543 patients in the Dara-VTd group achieved a stringent complete response (sCR), compared to 110 (20%) of 542 patients in the VTd group (odds ratio 1·60, 95% CI 1·21-2·12, *P* = 0.0010). Additionally, 211 (39%) patients in the Dara-VTd group versus 141 (26%) in the VTd group achieved a complete response or better, and 346 (64%) of 543 versus 236 (44%) of 542 achieved minimal residual disease-negativity (10^−5^ sensitivity threshold, assessed by multiparametric flow cytometry: both *P* < .0001). Median PFS from first randomization was not reached in either group (hazard ratio 0·47, 95% CI 0·33-0·67, *P* < .0001). After a median follow-up period of 45 months, the addition of daratumumab to induction showed improved PFS with a hazard ratio (HR) of 0.58 (95% confidence interval [CI]: 0.47-0.72). Although the data on overall survival are still premature, there were fewer deaths observed in the group receiving daratumumab (41 deaths compared to 73 deaths in the control group). This difference yielded an HR of 0.54 (95% CI: 0.37-0.79), a finding that holds potential clinical significance pending further confirmation during subsequent follow-up.

Another phase II randomized study found that daratumumab plus RVD (Dara-RVD) improved the rate and depth of response to therapy, and extended PFS in comparison to RVD.^53^ GRIFFIN study enrolled 207 patients who were randomly assigned to receive Dara-RVD or RVD induction (4 cycles), autologous HSCT, Dara-RVD or RVD consolidation (2 cycles), and lenalidomide or lenalidomide plus Dara maintenance (26 cycles). The primary endpoint, sCR rate at the end of post-HSCT consolidation, showed an improvement with Dara-RVD compared to RVD (42.4% vs 32.0%). With longer follow-up (median, 22.1 months), responses deepened; sCR rates improved for Dara-RVD vs RVD (62.6% vs 45.4%; *P* = .0177), as did minimal residual disease (MRD) negativity (10^−5^ threshold) rates in the intent-to-treat population (51.0% vs 20.4%, *P* < .0001). In the Dara-RVD group, the estimated 24-month PFS of 95.8%, while the RVD group exhibited a 24-month PFS rate of 89.8%. Hematologic AEs of grade III/IV were more common with Dara-RVD, and more infections occurred with Dara-RVD, although grade III/IV infection rates were similar.^53^ It is important to note that more patients who were assigned to control arm (RVD) dropped early from the study (<6 months after initiation) when compared to patients who received Dara-RVD which may be related to dissatisfaction with trial allocation. Long-term follow-up data as well as results from phase III trials comparing quadruplet to triplet therapies will be needed prior to making any definite conclusions; however, lack of long-term follow up is a common problem in clinical trials (Table 1).

While the benefit of daratumumab was more significant in standard-risk patients, both standard and high-risk disease showed a positive effect.^53,61^ Longer follow up data will be needed to assess OS benefit of quadruplet-based regimens. It is our practice to include anti-CD38 based therapy in transplant eligible MM patients when available for all patients regardless of disease risk given that most benefit was achieved for patients with standard risk disease.

Tandem autologous HSCT is our preferred strategy in high-risk MM given poor prognosis and lack of improvement over the years. A meta-analysis found that tandem autologous HSCT provided better survival rates compared to single HSCT (5-year OS, 70% vs. 17%; p < .001) for 606 patients with high-risk cytogenetics who did not achieve complete response after bortezomib-based induction.^62^ However, the STAMINA trial, which examined both tandem and single autologous HSCT, showed no difference in PFS or OS for patients with high-risk disease at 6 years in an intention-to-treat analysis. Nevertheless, in the as-treated analysis, patients with high-risk disease who received tandem autologous HSCT had better PFS than those who received single ASCT (43.6% vs. 26%; p = .03).^63,64^ In the EMN02/HO95 trial, there was no significant contrast in 5-year OS between patients with high-risk disease who received tandem autologous HSCT versus single HSCT (61.3% vs. 54.7%; p = .32). However, a subgroup of patients with del(17p) demonstrated a benefit with tandem autologous HSCT (80% vs. 57%; p = .066).^65,66^ It is important to notice that such different results may be related to different definitions of high-risk MM across various clinical trials.^67^

## Transplant Ineligibile

For patients newly diagnosed with MM who cannot undergo autologous HSCT, the primary initial therapy options are RD (lenalidomide and dexamethasone), bortezomib-based regimens (RVD), or anti-CD38-based regimens (Dara-Rd), or modified dosing of Dara-RVD. It is important to note that there is lack of comparative studies between Dara-Rd, RVD, and Dara-RVD. The decision is personalized based on patient and/or disease characteristics.

The use of melphalan-based regimens, such as VMP (bortezomib, melphalan, and prednisone), is considered only if there are problems with access to lenalidomide and it is not usually used in the US. Alternative alkylating agent such as cyclophosphamide can be used instead of melphalan to reduce AEs and will not affect stem cell mobilization and has more predictable dosing. A 4-drug regimen of Dara plus VMP has shown superior PFS and OS compared with VMP in a randomized phase III trial, but the contribution of the fourth drug to the induction component is unclear and this regimen is not usually used our practice.^68^

Our preferred regimen for transplant ineligible MM is Dara-Rd based on results from MAIA trial or a modified dosing of Dara-RVd for transplant-inelgibile patients with high-risk disease.^69^ MAIA study aimed to determine if the addition of daratumumab to the standard treatment of lenalidomide and dexamethasone would decrease the risk of disease progression or death in patients with newly diagnosed MM who were ineligible for autologous HSCT. The study involved 737 patients, and those in the daratumumab group had a lower risk of disease progression or death compared to those in the control group. After a median follow-up of 28.0 months (range, 0 to 41.4 months), disease progression or death was observed in 240 patients, with 97 out of 368 patients (26.4%) from the daratumumab group and 143 out of 369 patients (38.8%) from the control group. In the daratumumab group, the median PFS was not reached, while it was 31.9 months (95% CI, 28.9 to not reached) in the control group. The hazard ratio for disease progression or death was 0.56 (95% CI, 0.43 to 0.73; *P* < .001) in favor of the daratumumab group. The daratumumab group also had a higher percentage of patients with a complete response or better, and a higher incidence of neutropenia and pneumonia. Patients in the daratumumab group were more than 3 times as likely to be negative for MRD (10^−5^ threshold) than those in the control group (24.2% vs. 7.3%, *P* < .001).^65^ In an updated result, OS was better in patients treated with Dara-Rd.^70^

Although it is important to interpret cross-trial comparisons with caution, the results from the MAIA study compare favorably to those from the phase III SWOG S0777 study.^48,49^ A higher proportion of patients in the MAIA study was aged 65 years or older (99% in the MAIA study [median age 73 years] compared to 43% in SWOG S0777 [median age 63 years]). In addition, all patients in the MAIA study were ineligible for transplantation, whereas 31% of the patients in SWOG S0777 were not intended for future transplantation. Furthermore, patients in the bortezomib plus lenalidomide and dexamethasone group in SWOG S0777 received only 8 cycles of triplet therapy followed by lenalidomide and dexamethasone until disease progression, while patients in the daratumumab group in the MAIA study received triplet therapy until disease progression (Table 2).

**Table 2.** Selected triplet treatment regimens for transplant ineligible newly diagnosed multiple myeloma.

Clinical trial	Number of patients	Treatment regimen	Median age (years)	Median follow up (months)	Progression-free survival (months)	Overall survival (months)
ELOQUENT-1^71^	748	Elotuzumab + Rd vs Rd	73	65	31.4 vs 29.5	NR for both groups
TOURMALINE-MM2^72^	705	IRd vs Rd	73 vs 74	55	35.3 vs 21.8	NR vs 51.8
MAIA^69^	737	DRd vs Rd	73 vs 74	56	NR vs 34.4	NR in both groups
ENDURANCE^60^	1087	VRd vs KRd	65	9	34.4 vs 34.6	NR in both groups
SWOG-S0777^48,49^	525	VRD vs Rd	63	84	41 vs 29	NR vs 69

Abbreviations: Rd, lenalidomide, dexamethasone; IRd, ixazomib, lenalidomide, dexamethasone; DRd, daratumumab, lenalidomide, dexamethasone; VRd, bortezomib, lenalidomide, dexamethasone; KRd, carfilzomib, lenalidomide, dexamethasone; NR, not reached.

## Role of Maintenance Therapy

Maintenance/extended therapy in newly diagnosed MM patients provides prolonged disease control with potential benefit of achieving long-lasting remissions. The role of maintenance/extended therapy has been studied in both transplant and non-transplant eligible settings and showed promising results. Lenalidomide has been extensively studied as maintenance therapy post HSCT.^73^ A meta-analysis involving 1208 newly diagnosed MM patients showed median PFS of 52.8 months with lenalidomide maintenance therapy while OS was not reached.^74^ [Table 3]

**Table 3.** Selected phase III clinical trials on role of maintenance therapy in multiple myeloma.

Study	Patient population	Maintenance treatment	Median progression-free survival (months)
CALBG 100104^75,76^	TE-NDMM	R*	57.3
IFM 2005-02^77^	TE-NDMM	R*	41
Myeloma XI^78^	TE-NDMM	R*	57
FIRST^79^	TIE-NDMM	Rd vs Rd18	25.5 vs 20
SWOG S0777^80^	TIE-NDMM	Rd (VRd induction)	43
MAIA^70^	TIE-NDMM	Rd (DRd induction)	NR

^*^Post ASCT.

Abbreviations: TE-NDMM, transplant eligible newly diagnosed multiple myeloma; TIE-NDMM, transplant ineligible newly diagnoses multiple myeloma; R, lenalidomide; Rd, lenalidomide anddexamethasone; VRd, bortezomib lenalidomide and dexamethasone; DRd, daratumumab lenalidomide and dexamethasone; NR, not reached.

In non-transplant setting continues doublet or triplet regimens have been studied with resultant improvement in response.^81^ Most extensively studied drugs include lenalidomide, bortezomib, ixazomib, and daratumumab (Table 3). Bortezomib and ixazomib maintenance therapy in non-transplant eligible setting were associated with more AEs and are not commonly used. Use of ixazomib as maintenance therapy should be avoided given inferior outcomes compared to other available agents.

## Role of Minimal Residual Disease

Assessment of minimal residual disease (MRD) has garnered significant attention in recent years due to its association with improved outcomes when achieving MRD negativity.^82^ This trend has prompted investigations into the applicability of MRD in guiding decisions related to treatment intensification, consolidation, or deintensification, as evidenced by ongoing assessment in various clinical trials (Table 4).

**Table 4.** Selected studies evaluating the role of MRD to determine treatment.

Study	Patient population	Timing of MRD assessment	Technique
PERSEUS (NCT03710603)	TE-NDMM	1-2 years into MT	NGS
DRAMATIC (NCT04071457)	TE-NDMM	2 years post MT	NGS
MASTER (NCT03224507)	TE-NDMM	Post induction, ASCT, and consolidation	NGS
EMN 20 (NCT04096066)	TIE-NDMM	1-2 years of MT	NGS

Abbreviations: TE-NDMM, transplant eligible newly diagnosed multiple myeloma; TIE-NDMM, transplant ineligible newly diagnosed multiple myeloma; MT, maintenance treatment; ASCT, autologous stem cell transplant; NGS, next generation sequencing; MRD, minimal residual disease.

Nevertheless, challenges persist concerning the standardization, optimization of testing timing, and integration of MRD assessment into clinical practice. Notably, there is no universally accepted method for measuring MRD. Presently, next-generation sequencing (NGS) and next-generation flow (NGF) methods exhibit enhanced sensitivity. While both NGS and NGF demonstrate comparable sensitivity for detecting MRD within the overall population, NGS exhibits a heightened MRD positive detection rate and greater sensitivity in patients with complete response.^83^ This distinction becomes particularly evident through NGS’s superior power in predicting PFS and OS, as observed in the case of NGF.^84^

## Relapsed/Refractory Disease

Treatment of relapsed/refractory MM is becoming more complex and should be individualized. The International Myeloma Working Group defines refractoriness to a specific agent as relapse/progression while on treatment or within < 60 days from the last dose of the drug.^85,86^ However, these criteria do not fully capture the underlying biology of relapse or drug resistance and were developed at a time when treatment options were limited. It is important to personalize treatment decisions for patients with relapsed/refractory MM based on drug class- refractoriness rather than number of prior lines of therapies.

The choice of regimen depends on the patient’s response, and tolerance to prior therapies. It is debatable whether a switch to a different drug class is necessary at the time of relapse. The aggressiveness of the relapse, whether biochemical or clinical, determines the treatment approach, with biochemical relapses often managed by observation, dose adjustments or the addition of another agent, while aggressive relapses may require multiagent therapy. When selecting treatment, factors such as psychosocial issues, access to care, drug approvals, route of administration, and insurance coverage should be considered.

In most cases, it is advisable to combine 2 effective anti-myeloma medications along with steroids, if they are well tolerated.^87^ Lenalidomide-based therapy is frequently used as a first-line and maintenance treatment. The emergence of resistance to lenalidomide has now taken precedence as the key factor in determining salvage therapy selection. Although daratumumab is progressively being integrated into first-line treatments, patients who relapse while on daratumumab present a common challenge.^88,89^ Using drugs with new mechanisms of action can improve outcomes and overcome class resistance.^73,74^ For instance, for patients who have shown resistance to lenalidomide, a viable approach could be transitioning to a PI in conjunction with daratumumab and steroids. Alternatively, considering a switch to an alternative IMiD like pomalidomide is also a reasonable strategy, as it has demonstrated enhanced median PFS. For patients who did not receive anti-CD38 antibodies using daratumumab, carfilzomib, and dexamethasone based on CANDOR trial or isatuximab, carfilzomib, and dexamethasone based on IKEMA trial are reasonable options with excellent response rates^90,91^ (Table 5). Patients who are not resistant to lenalidomide experience benefits from the combination of daratumumab, lenalidomide, and dexamethasone, as evidenced by the findings of the POLLUX trial.^92,93^ Patients who progressed on both daratumumab and lenalidomide based initial treatment (DRd) can benefit from PI (bortezomib, carfilzomib)-based combination regimens, or pomalidomide-based regimens or cyclophosphamide-based regimens (Table 5).

**Table 5.** Select trials in relapsed/refractory multiple myeloma.

Clinical trial	Number of patients	Treatment regimen	Median age (years)	Median follow up (months)	Progression-free survival (months)	Overall survival (months)
CANDOR^91^	466	KdD vs Kd	64	27.8	28.6 vs 15.2	NR in both groups
ICARIA_MM^94^	307	Isa-Pd vs Pd	67	11.6	11.5 vs 6.5	NR in both groups
IKEMA^90^	302	Isa-Kd vs Kd	64	44	35.7 vs 19.1	NR in both groups
APOLLO^95^	304	Pom-Dara-Dex vs Pom-Dex	67	16.9	12.4 vs 6.9	NR in both groups
ELOQUENT-2^96,97^	646	Elotuzumab-Rd vs Rd	66	70.6	19.4 vs 14.9	48.3 vs 39.6
OPTIMISMM^98^	559	Pom-Vd vs Vd	67	15.9	11.2 vs 7.1	NR in both groups
POLLUX^92,93^	569	DRd vs Rd	65	54.8	45 vs 17.5	67.6 vs 51.8*
CASTOR^99^	498	D-Vd vs Vd	64	47	16.7 vs 7.1	49.6 vs 38.5∞

Abbreviations: KdD, Carfilzomib, dexamethasone, daratumumab; Kd, Carfilzomib, dexamethasone; Pom-Dex, Pomalidomide, dexamethasone; Isa-Pd, Isatuximab, pomalidomide, dexamethasone; Pd, pomalidomide, dexamethasone; Isa-Kd, Isatuximab, carfilzomib, dexamethasone; Pom-Dara-Dex, Pomalidomide, daratumumab, dexamethasone; Rd, Lenalidomide, dexamethasone; Pom-VD, pomalidomide, bortezomib, dexamethasone; Vd, Bortezomib, dexamethasone; NR, Not reached; DRd, Daratumumab, lenalidomide, dexamethasone; D-Vd, Daratumumab, bortezomib, dexamethasone.

*Median follow up: 79.7 months.

∞Median follow up: 72.6 months.

Patients who do not get front-line HSCT can benefit from salvage HSCT upon progression especially in standard risk patients where data from IFM 2009 and DETERMINATION trial did not show OS difference when HSCT was used as salvage treatment.^42,51^ One trial also showed benefit from second HSCT who had disease progression at least 12 months after first HSCT. The implementation of salvage HSCT improved OS when used to consolidate re-induction treatment for individuals with MM experiencing their first relapse subsequent to an initial HSCT.^100^ The International Myeloma Working Group does not recommend salvage HSCT in patients who progressed within 3 years of front-line HSCT.^101^

Promising advancements in relapsed/refractory MM include the utilization of chimeric antigen receptor (CAR) T-cell therapy and bispecific antibodies that target the B-cell maturing antigen (BCMA). These innovative approaches are exhibiting highly favorable initial outcomes among patients who have undergone extensive prior treatments. Furthermore, future clinical trials are exploring the potential of employing these methods at earlier stages of treatment and are comparing their efficacy against other active agents used for MM.

Teclistimab, a bispecific antibody designed to target BCMA, has demonstrated encouraging results in MM patients who have received substantial prior treatments. Specifically, it has shown a median PFS of 11.3 months and a median OS of 18.3 months in heavily treated MM cases.^102^ Notably, 2 recent phase III studies have showed that BCMA-targeted CAR T-cell therapy provides a PFS advantage compared to standard care treatments, as depicted in Table 6.

**Table 6.** Approved bispecific antibodies and chimeric antigen T-cell receptor therapy in multiple myeloma.

Clinical trial	Number of patients	Treatment regimen	Median age (years)	Median follow up (months)	Progression-free survival (months)
CARTITUDE-1^103^	97	Ciltacabtagene autoleucel	61	27.7	NR
KarMMa^104^	128	Idecabtagene vivleucel	61	15.4	8.8
CARTITUDE-4^105^	419	Ciltacabtagene autoleucel vs SOC	61	15.9	NR vs 11.8
KarMMa-3^106^	386	Idecabtegene vivleucel vs SOC	63	18.6	13.3 vs 4.4
MajesTEC-1^107^	125	Teclistimab CD3/BCMA	64	14.1	11.3
MagnetisMM-3^108^	123	Elranatamab CD3/BCMA	68	14.7	NR
MonumenTAL-1^109^	232	Talquetamab CD3/GPRC5D	62	11.7	7.5

Abbreviations: NR, not reached, SOC, standard of care, BCMA, B-cell maturation antigen, GPRC5D, G protein-coupled receptor family C group 5 member D.

**Figure 2.**
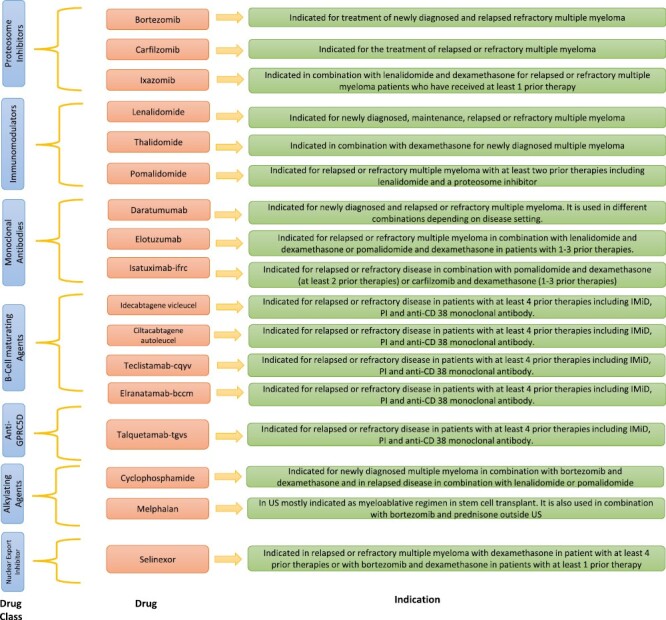
US FDA approved anti-myeloma drugs.

Nonetheless, the administration of CAR T-cell therapy poses a challenge due to the requirement for specialized facilities capable of managing potential AEs such as cytokine release syndrome and neurotoxicity. Despite this challenge, the encouraging data suggests that patients who have experienced disease progression after undergoing multiple prior treatments, HSCT, should be referred to specialized cellular therapy centers. Available classes of approved drugs are summarized in Fig. 2

## Areas of Unmet Need

Despite the significant progress made in the treatment of MM over the past 2 decades, there remain areas of unmet need in the management of this disease. One of the major challenges in the treatment of MM is the development of drug resistance. Although patients initially respond well to treatment, the disease often recurs and becomes resistant to the therapies used, ultimately leading to treatment failure and poor outcomes. Therefore, there is a need for new therapeutic strategies that can overcome drug resistance and provide durable responses.

Another area of unmet need in the treatment of MM is the management of bone disease. MM is associated with significant bone destruction, which can cause pain, fractures, and spinal cord compression, leading to disability and reduced quality of life. While bisphosphonates and denosumab have been shown to reduce skeletal-related events, they are not curative and do not restore bone integrity. Novel therapies that target bone remodeling and promote bone healing are needed to address this unmet need.

In addition, there is a need for more personalized approaches to the treatment of MM that will ensure appropriate treatment options are provided to patients while avoiding the risk of under/over treatment. While current therapies are effective for many patients, not all patients respond equally well to these treatments. Therefore, there is a need for better biomarkers and predictive tools that can identify patients who are most likely to benefit from a particular therapy and those who are at risk for treatment failure. This will allow for more targeted and individualized treatment approaches, which may improve outcomes and reduce the risk of toxicity.

## Importance of Clinical Trials

The approach to treating MM is continually advancing, with numerous newly approved drugs and several more currently in various stages of development and clinical trials. Ensuring our patients’ access to these innovative agents is of paramount importance, achieved through their participation in these trials. This holds particularly true for patients who have undergone extensive prior treatments and are faced with limited effective therapeutic choices. Enrolling such patients in clinical trials holds the potential not only to benefit them directly but also to contribute to the advancement of improved and more efficacious treatment strategies.

## Our Perspectives

Immunotherapy is a promising area for future directions in the treatment of MM. This includes the use of CAR T-cell therapy or bispecific antibodies. CAR T-cell therapy has shown promising results in clinical trials and is expected to become an important part of the treatment armamentarium for MM. Bispecific antibodies are available off the shelf and early results are showing excellent response rates and are quite promising and currently 3 products (teclistamab, elranatamab, and talquetamab) are approved in the U.S. Limited duration therapy clinical trials and focus on newer approaches to treat high-risk disease are needed. It is important to better identify the appropriate sequencing of available therapies to minimize AEs and get the maximum benefit of each treatment option. Finally, we believe that a fraction of patients with MM are cured with the use of intensive combinational therapy including IMiDs and PIs along with high-dose chemotherapy and HSCT^10^; however, efforts need to improve the fraction of cure while decreasing treatment intesity and/or AEs.

## Summary

The future for the treatment of MM is bright. The myeloma community needs to focus on improving treatment outcomes, overcoming drug resistance, and developing more personalized and targeted therapies. With continued research and innovation, it is hoped that we will be able to further improve the prognosis and quality of life for patients.

## Data Availability

No new data were generated or analyzed in support of this research.
